# Peroxisomal Dysfunction in Neurological Diseases and Brain Aging

**DOI:** 10.3389/fncel.2020.00044

**Published:** 2020-03-10

**Authors:** Ndidi-Ese Uzor, Louise D. McCullough, Andrey S. Tsvetkov

**Affiliations:** ^1^Department of Neurobiology and Anatomy, University of Texas McGovern Medical School, Houston, TX, United States; ^2^The University of Texas Graduate School of Biomedical Sciences, Houston, TX, United States; ^3^Department of Neurology, University of Texas McGovern Medical School, Houston, TX, United States; ^4^UTHealth Consortium on Aging, University of Texas McGovern Medical School, Houston, TX, United States

**Keywords:** neuronal peroxisomes, peroxisome biogenesis disorders, aging peroxisomes, neurodegenerative disease, peroxisomal dysfunction

## Abstract

Peroxisomes exist in most cells, where they participate in lipid metabolism, as well as scavenging the reactive oxygen species (ROS) that are produced as by-products of their metabolic functions. In certain tissues such as the liver and kidneys, peroxisomes have more specific roles, such as bile acid synthesis in the liver and steroidogenesis in the adrenal glands. In the brain, peroxisomes are critically involved in creating and maintaining the lipid content of cell membranes and the myelin sheath, highlighting their importance in the central nervous system (CNS). This review summarizes the peroxisomal lifecycle, then examines the literature that establishes a link between peroxisomal dysfunction, cellular aging, and age-related disorders that affect the CNS. This review also discusses the gap of knowledge in research on peroxisomes in the CNS.

## Introduction

**Figure 1:**
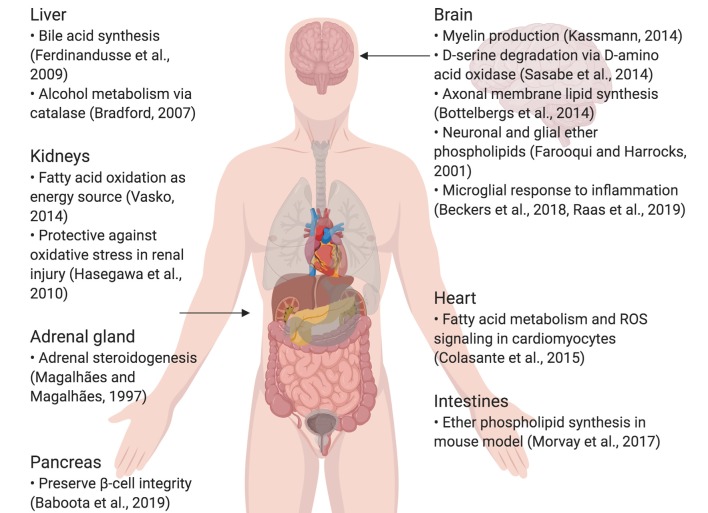
Summary of specialized roles of peroxisomes in some organs, including the brain. Created using BioRender.

Peroxisomes are small, nearly ubiquitous organelles found in almost all cell types, except mature red blood cells (Gronowicz et al., 1984). Their major functions include the beta-oxidation of very-long-chain fatty acids and lipid peroxidation; as a result of this metabolism, they secrete reactive oxygen species (ROS) as by-products (Reddy and Hashimoto, 2001; Poirier et al., 2006; Lodhi et al., 2015; Park et al., 2019). Peroxisomes also possess enzymes that break down ROS, such as catalase and glutathione peroxidase, which breaks down hydrogen peroxide, and superoxide dismutase, which breaks down superoxide (Nordgren and Fransen, 2014). They degrade prostaglandins, amino acids, polyamines, and purines, and are commonly enriched in the kidneys, liver, pancreas and adrenal glands, which are involved in fat metabolism and detoxification (Magalhães and Magalhães, 1997; Bradford, 2007; Ferdinandusse et al., 2009; Hasegawa et al., 2010; Smith and Aitchison, 2013; Vasko, 2016; Baboota et al., 2019). Furthermore, they are implicated in lipogenic and ROS signaling roles in the heart and intestines (Colasante et al., 2015; Morvay et al., 2017). In the central nervous system (CNS) in particular, peroxisomes synthesize lipids that make up the myelin sheath and cellular membranes, as well as ether phospholipids in neurons and glia; peroxisome dysfunction is also known to impair neuronal migration and membranes (Farooqui and Horrocks, 2001; Powers, 2001; Bottelbergs et al., 2010; Kassmann, 2014). They also play a critical role in breaking down D-serine *via* D-amino acid oxidase (DAO), important in glutamatergic signaling (Sasabe et al., 2014; Figure 1). Certain diseases, such as peroxisomal biogenesis disorders, underscore the importance of functional peroxisomes in the CNS. Peroxisomal biogenesis disorders are a subset of diseases where: (1) peroxisomes are either not present, leading to severe neurological phenotypes (as seen in neonatal adrenoleukodystrophy, where seizures, hypotonia, and loss of vision and hearing occur) and a short lifespan; or (2) genes coding for a single peroxisomal protein are defective, where the symptoms are not as severe (Fujiki et al., 2012; Aubourg et al., 2013). To conclude, peroxisomes are small, but important organelles that play supportive, yet critical roles in maintaining cellular health, especially in the CNS.

This review summarizes peroxisomal biogenesis, and yeast and mammalian pexophagy, with an extended focus on peroxisomes in cellular senescence models, and the peroxisomal dysfunction shared by both age-related neurodegenerative diseases and peroxisomal biogenesis disorders. In all of these conditions, functional peroxisomes move from understudied, secondary organelles to critical sustainers of cellular homeostasis that are disrupted by disease. Future studies will elucidate the role of peroxisomes in aging and CNS function in other diseases and models.

## Peroxisomal Biogenesis

Peroxisomes begin their lifecycle by budding off the endoplasmic reticulum in response to peroxisome proliferator-activated receptor (PPAR) activation due to signaling of the PPAR gamma coactivator-1α (PGC-1α) protein (Bagattin et al., 2010). Unlike mitochondrial proteins, peroxisomal proteins are synthesized on free ribosomes in the cytosol (Koehler, 2000; Jan et al., 2014). After this, peroxisomal proteins are inserted into peroxisomal membranes and matrices by the peroxisomal protein Pex5 (Smith and Aitchison, 2013). Pex5 recognizes the peroxisomal targeting sequence (PTS1) serine-lysine-leucine (SKL), which is found on the C-terminal of many peroxisomal proteins (Brocard and Hartig, 2006). After proteins are inserted, peroxisomes are considered mature and functional. For peroxisomal maintenance, division and maturation, peroxisomes are known to make contact with the endoplasmic reticulum (Hua et al., 2017). To conclude, peroxisomal division and maintenance are modulated by the endoplasmic reticulum, and peroxisomes mature due to peroxisomal protein import into their matrices and membranes.

## Autophagy and Pexophagy

**Figure 2:**
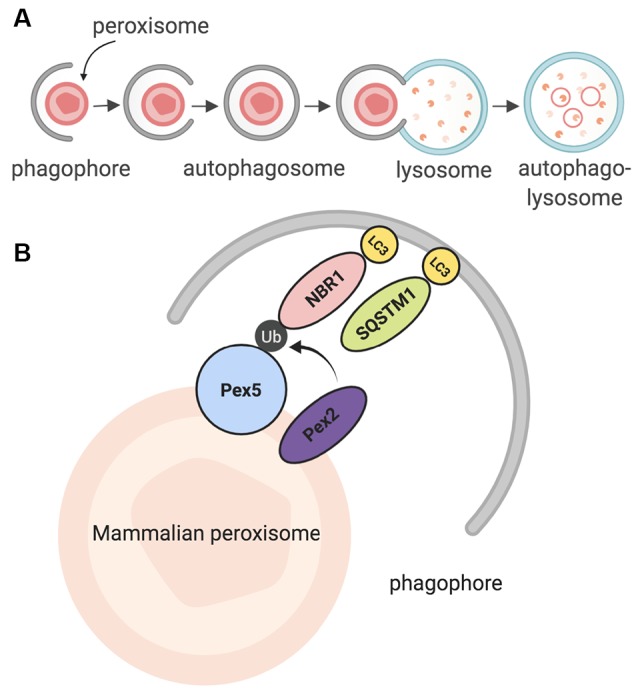
Summary of pexophagy. **(A)** In macropexophagy, a form of macroautophagy selective for peroxisomes, a single membrane known as a phagophore engulfs a peroxisome for degradation. The phagophore matures into an autophagosome, which then fuses with a lysosome. Their fusion creates an autophagolysosome that degrades the target. **(B)** In mammalian systems, pexophagy occurs when Pex2 ubiquitinates Pex5. As a result, autophagy adaptor proteins NBR1 or SQSTM1 (p62) bind to ubiquitinated Pex5, and then eventually bind to LC3 on the phagophore. Due to this process, pexophagy occurs. Created using BioRender.

The peroxisomal lifespan in mammalian cells lasts about 2 to 3 days (Poole et al., 1969; Huybrechts et al., 2009; Moruno-Manchon et al., 2018b). Peroxisomes are then degraded by a selective form of macroautophagy: macropexophagy, which specifically targets peroxisomes (Yang and Klionsky, 2010; Bartoszewska et al., 2012; Cho et al., 2018). A lesser-known form of pexophagy micropexophagy exists, but has only been, so far, observed in yeast models (Strømhaug et al., 2001; Mukaiyama et al., 2004). In macroautophagy, targets for degradation are recognized by a phagophore, which matures to form an autophagosome (Reggiori and Tooze, 2009; Mizushima et al., 2011; Feng et al., 2014; Biazik et al., 2015; Moruno Manchon et al., 2015, 2016). The autophagosome envelops the targets and then fuses with an acidic structure known as the lysosome. Together, they form the autophagolysosome, which degrades the target (Figure 2A; Nakamura and Yoshimori, 2017; Sasaki et al., 2017). Pexophagy itself uses the same process; however, peroxisomes are targeted *via* particular proteins on their membrane (Jin et al., 2013; Cho et al., 2018). Once recognized, peroxisomes are enveloped by the phagophore, and eventually degraded by the autophagolysosome. Recently, a study in HeLa SH-SY5Ycells and mutant *Drosophila* flies unearthed a novel pexophagy inducer: HSPA9, a heat shock protein which responds to cellular changes such as glucose deprivation (Jo et al., 2020). In summary, peroxisomes that have reached the end of their life cycle are degraded through a selective autophagic process known as pexophagy, due to the enzymatic action of the autophagolysosome.

## Pexophagy in Mammals

The foundation of the pexophagy mechanism (and peroxisome biology) began in studies using yeast and plants as models (Lefevre et al., 2013; Williams and van der Klei, 2013; Kao and Bartel, 2015; Young and Bartel, 2016). The *Saccharomyces cerevisiae* yeast homolog of PEX5, Pex5, recognizes cytosolic peroxisomal matrix proteins and delivers them to the peroxisomal membrane (Carvalho et al., 2007). In this model, pexophagy involves Pex3, which is recognized and bound by phosphorylated Atg36, which is itself recognized by Atg8 or Atg11, which are bound to the phagophore (Motley et al., 2012a,b; Farré et al., 2013; Yamashita et al., 2014). In some cases, such as when mitochondria and peroxisomes interact, peroxisomal fission occurs before pexophagy, modulated by Dnm1 and Vps1 (Mao et al., 2014).

Further studies in mammalian models revealed that in order for mammalian pexophagy to begin, PEX5 has to be monoubiquitinated by PEX2, an E3 ubiquitin ligase (Nordgren et al., 2015; Sargent et al., 2016; Germain and Kim, 2020). Previously, it was not clear what directly induces pexophagy; however, recent evidence has shown that increased ROS in the cytosol can stimulate this monoubiquitination, allowing PEX5 to act as a ROS sensor, leading to an increase in peroxisome degradation (Kim et al., 2008; Zhang et al., 2015; Walton et al., 2017). After PEX5 is monoubiquitinated, it is recognized by one of two LC3 adaptor proteins: NBR1, or p62 (SQSTM1); these proteins are then bound to LC3, which is bound to the autophagosome (Figure 2B; Kabeya et al., 2000; Kirkin et al., 2009; Deosaran et al., 2013). Together, the interaction of these proteins induces pexophagy in the mammalian cell.

## The Known: Dysfunctional Peroxisomes and Pexophagy in Neurodegenerative Disease, Peroxisomal Disorders, and Neuropathies

In the CNS, neurons rely on different forms of autophagy (general and selective) to clear organelles and proteins that are no longer of use; this use of autophagy is due to neurons being post-mitotic and unable to divide, making them more vulnerable than cells that can divide and dilute toxic protein build-up (Moore and Holzbaur, 2016; Evans and Holzbaur, 2019; Stavoe and Holzbaur, 2019a,b). Neuronal autophagy is compartment-specific: it begins at the distal axon, after which axonal autophagosomes then move into the cell soma; the soma also contains its own autophagosomes (Maday and Holzbaur, 2016; Kulkarni et al., 2018; Moruno-Manchon et al., 2018a). Neurons also respond to autophagy inducers differently than other neural cells, underscoring the uniqueness of neuronal autophagy among other forms of autophagy (Ferguson et al., 2009; Pamenter et al., 2012; Bordi et al., 2016; Moruno Manchon et al., 2016; Kulkarni et al., 2019; Sung and Jimenez-Sanchez, 2020).

Interestingly, a common trait of neurodegenerative diseases is the impairment of protein and organelle turnover. Alzheimer disease (AD) is the most common form of dementia in elderly people, with patients exhibiting symptoms such as memory loss and mood changes; the disease eventually destroys neurons in the hippocampus and the cortex (Liang et al., 2008; GBD 2013 Mortality and Causes of Death Collaborators, 2015). In AD, beta-amyloid and tau accumulate, and senescent mitochondria are also present (Zilka et al., 2006; Mitchell, 2009; Nilsson et al., 2013; Shi et al., 2016; Harada et al., 2018). While Parkinson’s disease (PD) has a lower prevalence than AD, the number of people with PD has increased over time, as the number of aged people has increased (Dorsey et al., 2018). In PD, neurodegeneration occurs in the substantia nigra, leading to tremors, bradykinesia, postural instability, and rigidity (Jagadeesan et al., 2017). Huntington disease (HD) occurs due to the mutated huntingtin gene and affects the medium spiny neurons in the striatum as well as neurons in the cortex, leading to symptoms such as chorea (jerky movements), rigidity and progressive motor failure (Ehrlich, 2012; Wyant et al., 2017). In Parkinson disease and HD, damaged mitochondria and causative proteins (alpha-synuclein and to a much smaller extent, tau in PD, and mutant huntingtin in HD) accumulate in affected neurons, indicating a problem with autophagy or the ubiquitin/proteasome system (Bloom, 2014; Atik et al., 2016; Zhao et al., 2016; Chiasseu et al., 2017; Zhang et al., 2018; Finkbeiner, 2019; Harrison et al., 2019). Amyotrophic lateral sclerosis (ALS) can be familial or sporadic, leading to neurodegeneration of motor neurons in the CNS; a wide range of genetic mutations can induce this neurodegeneration, including the *SOD1* gene, which codes for superoxide dismutase (Peters et al., 2015). Inducing autophagy improves survival in neuronal ALS models (Barmada et al., 2014). In aging neurons, mitochondrial senescence is observed (Gilmer et al., 2010; Menzies et al., 2017). However, not much is known about how pexophagy, or how peroxisomal proteins are affected by these diseases. First, we will summarize the present data on peroxisomes and pexophagy in neurodegenerative disease studies, then review cases where global peroxisomal disturbances lead to neurodegenerative phenotypes.

In some neurodegenerative diseases, the amount and/or function of peroxisomes may be compromised. In Alzheimer’s disease, in which beta-amyloid and tau accumulate in neurons, peroxisomes may be affected. In one study, rat hippocampal cultures with beta-amyloid overexpression were treated with Wy-14.463, a peroxisomal proliferator. This treatment increased peroxisomal number and catalase activity reduced ROS production, and overall, reduced the degenerative effects of beta-amyloid such as the instability of beta-catenin and the increase of calcium (Santos et al., 2005). In a clinical study, plasmalogens (which peroxisomes synthesize) were negatively affected in post-mortem samples of Alzheimer patients’ brains, suggesting a reduction in peroxisomal activity, or a shorter half-life of plamalogens (Goodenowe and Senanayake, 2019). ALS, a disease in which motor neurons degenerate, is linked to peroxisome dysfunction through a genetic mutation that codes for DAO, a peroxisomal enzyme that specifically breaks down D-serine (Kondori et al., 2017, 2018).

In other cases, peroxisome dysfunction, as seen in peroxisome biogenesis disorders, may lead to degenerative neurological symptoms. Peroxisome biogenesis disorders occur due to peroxisome genetic defects, either resulting in single peroxisomal enzyme dysfunction, or in rare cases, the absence of peroxisomes themselves (Braverman et al., 2016). Two groupings of peroxisome biogenesis disorders exist under the Zellweger spectrum (neonatal adrenoleukodystrophy, Zellweger syndrome and infantile Refsum disease), and those outside of it. In Zellweger syndrome, which is inherited in an autosomal recessive manner, one of 13 peroxin (*PEX*) genes is mutated (*PEX1*, *PEX2*, *PEX3*, *PEX5*, *PEX6*, *PEX10*, *PEX11β*, *PEX12*, *PEX13*, *PEX14*, *PEX16*, *PEX19*, *PEX26*), leading to issues with neuronal migration, myelination and brain development (Waterham and Ebberink, 2012; Klouwer et al., 2015; Wang et al., 2015). A cellular model of Zellweger syndrome, particularly of a *Pex5* mutation, has shown an increase in alpha-synuclein Lewy bodies; alpha-synuclein is thought to be a causative agent in Parkinson disease, particularly in familial cases (Yakunin et al., 2010; Riederer et al., 2019). *In vivo*, *Pex5*^−/−^ mouse brain samples exhibited an increase in alpha-synuclein oligomers in comparison to control, suggesting a correlation between peroxisome dysfunction and PD (Yakunin et al., 2010). Neonatal adrenoleukodystrophy is also an autosomal recessive PBD, but with multiple peroxisomal enzymes affected; infant patients exhibit neurological symptoms such as hearing loss, neuropathy, and demyelination (Aubourg et al., 1986). The last PBD under the Zellweger spectrum is infantile Refsum disease, where a build-up of phytanic acid and other very-long-chain fatty acids in the body (a result of mutated *PEX* genes) leads to neurological symptoms such as mixed neuropathy and hearing loss (Warren et al., 2018). Outside the Zellweger spectrum, adult Refsum disease has similar symptoms to infantile Refsum disease, but the adult-onset disease is due to a mutation in the *PHYH* gene that codes for the peroxisomal enzyme phytanoyl-CoA dioxygenase, which peroxisomes use to break down phytanic acid into pristanic acid (Wanders et al., 2011; Wanders and Poll-The, 2017; Gettelfinger and Dahl, 2018).

**Figure 3:**
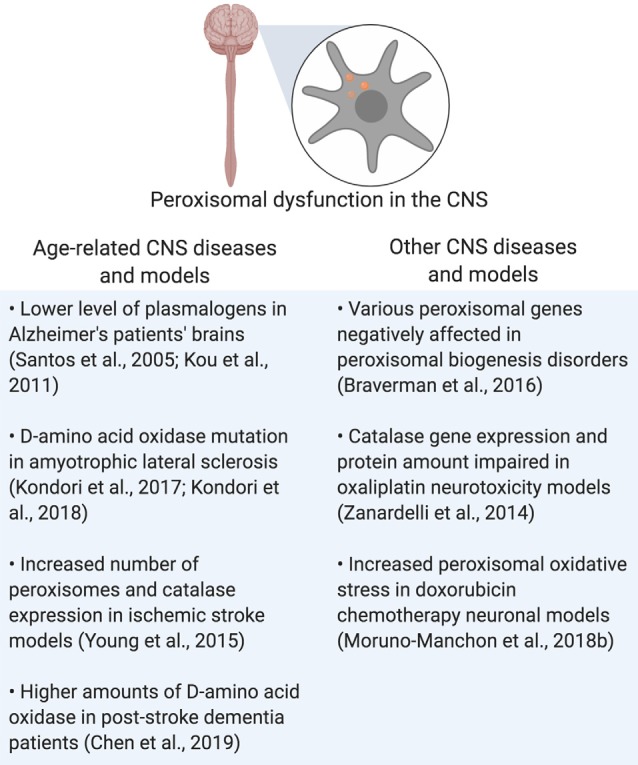
Summary of peroxisomal dysfunction in age-related diseases in the CNS, and other diseases that affect the CNS. Created using BioRender.

Rhizomelic chondrodysplasia punctata (RCDP) is a set of peroxisome biogenesis disorders where peroxisomal genes coding for proteins involved in plasmalogen synthesis are mutated (Barøy et al., 2015). Of note is RCDP type 1, which is due to the mutation of *PEX7*, which codes for PEX7, a peroxisomal receptor that inserts proteins into the peroxisomal membrane that carries peroxisome targeting signal 2 (PTS2; Purdue et al., 1999). This mutation results in severe neurological symptoms such as epilepsy and age-related conditions such as cataracts (Purdue et al., 1999; Malheiro et al., 2015; Landino et al., 2017). In conclusion, peroxisomal dysfunction in the CNS is shared by both neurodegenerative and peroxisomal disorders, leading to disrupted cellular homeostasis that contributes to the pathogenesis of those diseases (Figure 3 and Table 1).

**Table 1:** Summary of neurological symptoms in neurological and peroxisomal disorders that arise as a result of peroxisomal dysfunction.

Neurological disorder	Peroxisomal protein/function affected	Neurological result
Alzheimer disease	Plasmalogen production	Lowered plasmalogens in the brain, increase in peroxisomal density and VLCAS in gyrus frontalis; peroxisome loss correlated with tau (Santos et al., 2005; Kou et al., 2011)
Amyotrophic lateral sclerosis (ALS)	D-amino acid oxidase (DAO) enzyme	DAO inactivity; increase in D-serine (Kondori et al., 2017, 2018)
Oxaliplatin neuropathy models	Catalase expression and amount	Lipid peroxidation; neuropathic phenotype in an animal model (Zanardelli et al., 2014)
Post-stroke dementia	D-amino acid oxidase (DAO) enzyme	Increase in DAO in patient plasma levels (Chen et al., 2019)∣rule
**Peroxisomal disorder**	**Peroxisomal gene affected**	**Neurological result**
Adult Refsum disease	*PHYH*	Phytanic acid buildup, anosmia, polyneuropathy, hearing and vision loss (Wanders et al., 2011; Wanders and Poll-The, 2017; Gettelfinger and Dahl, 2018)
Infantile Refsum disease	*PEX1*, *PEX3*, *PEX6*, *PEX12*, *PEX26*	Phytanic acid buildup, hypomyelination, hearing and vision loss, polyneuropathy (Warren et al., 2018)
Neonatal adrenoleukodystrophy	*PEX1, PEX2, PEX3, PEX5, PEX6, PEX10, PEX11β, PEX12, PEX13, PEX14, PEX16, PEX19, PEX26*	Buildup of VLCFAs, seizures, hearing loss, neuropathy (Aubourg et al., 1986)
Rhizomelic chondrodysplasia punctata	*PEX7; PEX5* (short isoform)	Epilepsy, seizures, cataracts, neuroregression (Purdue et al., 1999; Malheiro et al., 2015; Landino et al., 2017)
Zellweger syndrome	*PEX1, PEX2, PEX3, PEX5, PEX6, PEX10, PEX11β, PEX12, PEX13, PEX14, PEX16, PEX19, PEX26*	Limited neuronal migration, issues with myelination and brain development (Waterham and Ebberink, 2012; Klouwer et al., 2015)

Peroxisomal dysfunction also contributes to neuropathies. For instance, oxaliplatin, a chemotherapy drug for colorectal cancer, is known to cause peripheral neuropathies in patients (Grothey, 2003; Banach et al., 2018). A study uncovered the role of peroxisomes in this mechanism using primary rat astrocyte cultures, a human colon cancer cell line and *ex vivo* analysis of an oxaliplatin neuropathy rat model: peroxisomal catalase expression and levels were impaired with oxaliplatin treatment of cell cultures, and in the dorsal root ganglia and spinal cords of treated animals; this change was also linked with lipid peroxidation in the spinal cord of treated animals (Zanardelli et al., 2014). More recent research has strengthened the role of peroxisome function in neuropathies: the peripheral nerves in peroxisomal mutation mouse models exhibited various abnormalities, such as impaired lysosomal function, accumulation of ganglioside, and a changed redistribution of Kv1 channels and their anchoring proteins that may lead to impaired signaling (Kleinecke et al., 2017). These studies, in conclusion, highlight the important, but previously hidden role that peroxisomal function plays, not only in the CNS but in peripheral nerves as well.

## The Somewhat Known: Cellular Biology of Peroxisomes in Neural Cell Types

As previously mentioned, peroxisomes are negatively affected by disorders that affect the CNS, leading to undesirable consequences. Some characterization of basal peroxisomal pathways has been made in oligodendrocytes and astrocytes in the CNS (Chistyakov et al., 2014; Di Cesare Mannelli et al., 2014; Aguirre-Rueda et al., 2015; Nury et al., 2018). In the case of neurons, there has also been a focus on peroxisomes (Ballister et al., 2015; Olenick et al., 2016). In hippocampal neurons, it was discovered that preventing tuberous sclerosis complex 2 (TSC2; a regulator of mTORC1 activity) from localizing to peroxisomes led to several axons extending from the neuronal body, indicating a change in morphology (Zhang et al., 2013). In studies of noise-induced hair loss, neurons in mice deficient in pevjakin (a protein associated with neuronal peroxisomes in the auditory pathway), exhibited less peroxisomal proliferation in response to loud sounds in comparison to their wild-type counterparts; peroxisomal proliferation is protective against oxidative stress produced by loud sounds (Defourny et al., 2019). We recently discovered that in neuronal models of doxorubicin treatment (a chemotherapy drug that leads to chemobrain), peroxisomes exhibited increased oxidative stress, which eventually damaged neurons (Kesler, 2014; Wefel et al., 2015; Kesler and Blayney, 2016; Manchon et al., 2016; Moruno-Manchon et al., 2016, 2018b). A more positive link has been found between peroxisomes and ischemic stroke; peroxisomal volume in *in vitro* and *in vivo* models of ischemia increased after injury, leading to an increased number of peroxisomes, as well as increased expression of peroxisomal catalase (Young et al., 2015). Inhibiting catalase or dynamin-related protein 1 (Drp1), a protein needed for peroxisomal fission, led to increased neuronal susceptibility to death from oxygen-glucose deprivation (OGD), a cellular model of ischemic stroke (Young et al., 2015). These findings inspired a clinical study, which investigated the link between post-stroke dementia (PSD) and peroxisomal DAO, an enzyme that oxidizes D-serine; plasma levels of PSD patients had higher levels of DAO, indicating its role in stroke and stroke-related dementia (Chen et al., 2019). In conclusion, these neuronal studies show that peroxisomal dysfunction can contribute to changes in neuronal morphology, increased oxidative stress, and even death in the CNS. Therefore, it is crucial to keep the negative side effects of treatments on various metabolic pathways, including those that involve peroxisomes, in mind.

## The Somewhat Known: Cellular Biology of Peroxisomes in Microglia

The link between peroxisomal function and inflammation has been established in non-CNS models; however, a few microglial studies have shed light on potential peroxisomal dysfunction mechanisms in the brain (Di Cara et al., 2019). For one, deleting the MFP2 peroxisomal enzyme (which is responsible for β-oxidation) in mouse microglia, switched their state to a pro-inflammatory one, but this change did not affect neuronal health or the microglial response to injury (Beckers et al., 2019). Another study looked at a neuron-specific form of MFP2 deletion and discovered that unlike constitutive *Mfp2^−/−^* knockouts, *Nestin-Mfp2^−/−^* knockout brains possessed microglia that were not primed for an inflammatory response (Beckers et al., 2018). Microglial peroxisomal dysfunction, as seen in a microglial model deficient in acyl-CoA oxidase 1 (ACOX1), has also been shown to affect catalase activity, the peroxisome, lipid droplet and mitochondrial number in microglia, as well as the induction of interleukin-1β (IL-1β), the repression of interleukin-6 (IL-6) and the increased expression of *Trem2*, which codes for a cell surface protein that plays a role in microglial phagocytosis (Raas et al., 2019). Taking these studies together, it can be assumed that microglial peroxisomal dysfunction affects the inflammatory response of microglia in the brain, directly and indirectly. The results of these microglial studies stress the importance of the peroxisomal role in inflammation of the CNS: peroxisomal dysfunction in microglia may lead to a pro-inflammatory response that negatively affects the whole system.

## The Unknown: Peroxisomes in the Normal Aging CNS

Nonetheless, one gap in the literature exists regarding peroxisomes in the aging CNS, that is unaffected by neurodegenerative disease. Non-neuronal senescence studies have shed some light on peroxisomes in aging cells, such as in senescent human fibroblasts, where there is a reported reduction in the import of PTS1-tagged proteins, an increase in hydrogen peroxide and peroxisomal number, and changes in peroxisomal appearance (Legakis et al., 2002). Proteomic analysis of *C. elegans* also shows a reduction of peroxisomal protein import, as well as a reduction in about 30 peroxisomal proteins, including PRX-5, the nematode homolog of PEX5; PRX-5 was also found to be mislocalized in the aged animals, suggesting that peroxisomal proteins were not properly localized (Narayan et al., 2016). Knocking it out reduced brood size, implicating a potential role of PRX-5 in both development and aging (Narayan et al., 2016). Cell type-specific ribosome profiling of *Drosophila melanogaster* oenocytes (cells involved in liver-like processes) revealed that peroxisomal pathways were downregulated with aging (Huang et al., 2019). Some related evidence exists in post-mortem Alzheimer’s studies, where there is an increase in peroxisomal density and very-long-chain fatty acids (but a reduction in plasmalogen levels) in neurons in the gyrus frontalis of AD patients, and a loss of peroxisomes in neuronal processes where phosphorylated tau is present (Kou et al., 2011). However, a search of the literature does not currently reveal evidence of peroxisomal perturbations in the normal aging brain. Another gap in the literature is present when investigating how sex, particularly in age-related neurological disease, affects peroxisomes. For instance, there is evidence that a sex difference exists in response to cerebral ischemia, or ischemic stroke, but it is unknown how these sex-associated differences may affect peroxisomes specifically (Siegel and McCullough, 2013; Mirza et al., 2015; Ritzel et al., 2017). Future studies on age-related neurological changes should investigate how peroxisomal pathways are affected, given the important roles that peroxisomes play in the brain, and how they are affected in other related diseases.

## Conclusion

As small and understudied as they are, there is ample evidence that peroxisomes play a supportive, yet critical role in the maintenance of the CNS; future studies should investigate the treatment of neurological diseases while keeping the peroxisomal role in maintaining cellular homeostasis in mind.

## Author Contributions

N-EU wrote the manuscript. All authors contributed to manuscript revision and references, read and approved the submitted version.

## Conflict of Interest

The authors declare that the research was conducted in the absence of any commercial or financial relationships that could be construed as a potential conflict of interest.
